# Training Specificity for Athletes: Emphasis on Strength-Power Training: A Narrative Review

**DOI:** 10.3390/jfmk7040102

**Published:** 2022-11-16

**Authors:** Michael H. Stone, W. Guy Hornsby, Dylan G. Suarez, Marco Duca, Kyle C. Pierce

**Affiliations:** 1 Center of Excellence for Sport Science and Coach Education, Department of Sport, Exercise, Recreation and Kinesiology, East Tennessee State University, Johnson City, TN 37614, USA; 2 School of Sport Sciences, College of Applied Human Sciences, West Virginia University, Morgantown, WV 26505, USA; 3 Sports Science, Cincinnati Reds, Cincinnati, OH 45202, USA; 4 Department of Kinesiology and Health Science, Louisiana State University Shreveport, Shreveport, LA 71115, USA

**Keywords:** strength endurance continuum, dynamic correspondence, programming methods

## Abstract

Specificity has two major components: A strength-endurance continuum (S-EC) and adherence to principles of Dynamic Correspondence. Available evidence indicates the existence of the S-EC continuum from two aspects. Indeed, the S-EC exists, particularly if work is equated as a high load low repetition scheme at one end (strength stimulus) and high volume (HIEE stimulus) at the other. Furthermore, some evidence also indicates that the continuum as a repetition paradigm with high-load, low repetition at one end (strength stimulus) and a high repetition, low load at the other end. The second paradigm is most apparent under three conditions: (1) ecological validity—in the real world, work is not equated, (2) use of absolute loads in testing and (3) a substantial difference in the repetitions used in training (for example 2–5 repetitions versus ≥10 repetitions). Additionally, adherence to the principles and criteria of dynamic correspondence allows for greater “transfer of training” to performance measures. Typically, and logically, in order to optimize transfer, training athletes requires a reasonable development of capacities (i.e., structure, metabolism, neural aspects, etc.) before more specific training takes place.

## 1. Introduction

Resistance training has been shown to improve a variety of performance and health-related variables [1,2,3,4,5,6,7]. Resistance training enhancement of performance related variables can include increased maximum strength, rate of force development, power, and both low- (LIEE) and high-intensity (HIEE) exercise endurance [8,9,10,11,12,13]. Changes in these variables (strength, power, etc.) as a result of resistance training have been associated with improved measures of athletic performance, such as the vertical jump, rugby tackling, sprint times, distance-running times, and change of direction [10,12,14,15,16,17,18,19]. Programs incorporating strength training as an integral part of physical conditioning have also been shown to improve performance in daily and ergonomic tasks, such as lifting weighted boxes to different heights [20,21]. These observations indicate that resistance training can have a substantial transfer–of-training effect that results in a change in “functional” ability and capacity. Underlying mechanisms supporting alterations in performance can include, cardiovascular and microvascular alterations, increased lean body mass and decreased fat; increased skeletal muscle CSA, particularly for the type II:I ratio; increased tissue tensile strength, including bone, and decreased physiological stress [5,22,23,24,25,26,27,28]. Indeed, choosing appropriate training methods (periodization, programming) can make a considerable difference in the outcome of a resistance-training process [15,16,22,29,30,31,32]. For example, high-volume programs, to a point [33], have a greater influence on muscle CSA, body composition, health and endurance factors than do low-volume programs [5,34,35,36]. Evidence also indicates that the choice of training mode (type of equipment) can influence the adaptations to a training program [37,38,39].

These findings clearly indicate that resistance-induced training alterations can substantially transfer to other aspects of sport and daily activities as well as enhance health. It appears that alterations of physical capabilities occur as a result of the primary components of training programs, the volume (work performed), intensity (the rate of ATP use) and frequency of exercise sessions. Indeed, Hawley et al. [40] indicate that these ‘training impulses’ are determinates of the magnitude of adaptive responses that can enhance or decrease exercise ability and the development of fitness capacities. However, the degree of “transfer of training effect” can depend upon the training principle *Specificity.* The primary aim of this paper is to examine the characteristics of the specificity concept. This includes a re-examination of the *Strength-Endurance Continuum* and a further examination of the of *Dynamic Correspondence* paradigm. This characterization can allow athletes and coaches to make superior choices in designing training programs.

## 2. Methods

Literature was gathered from Google Scholar, Pubmed and Researchgate. Literature was confined mainly to those studies using free weights and considered strength and high intensity exercise endurance (HIEE). Key words and phrases (English) used in the search included, strength, endurance, strength-endurance, power endurance, strength—endurance continuum, training to failure and high intensity exercise. HIEE is a term denoting exercise in which the primary bioenergetic mechanisms are phosphogens and fast (anaerobic) glycolysis. As HIEE deals with near maximum and maximum rates of ATP production, HIEE may take the form of repetitions to failure, particularly using complex exercises such as squats or as high peak or average power outputs such as with Wingate tests. Furthermore, the review largely deals with alterations in athletic related performance.

## 3. Specificity

Specificity concerns the degree of bioenergetics and biomechanical similarity between training modes and methods, and performance. Indeed, there is little doubt that, as a result of genetics and training for long periods in different manners, adaptations and capabilities impacting strength and endurance related parameters are markedly different [41,42,43]. Substantial differences can occur even among strength-power athletes using resistance training in different manners [44,45,46]. Thus, it appears that specificity is a primary factor, dictating the types of physiological and biomechanical adaptations resulting from defined stimuli [47,48,49] as well as specific performance outcomes [50]. However, overload, represented by training impulses and the development of “capacities,” seems to be at odds with the concept of specificity [51].

Conceptually, specificity primarily depends upon the existence of two conceptual paradigms, the Strength- Endurance (S-E) continuum and the idea of Dynamic Correspondence (DC).

**Figure 1:**
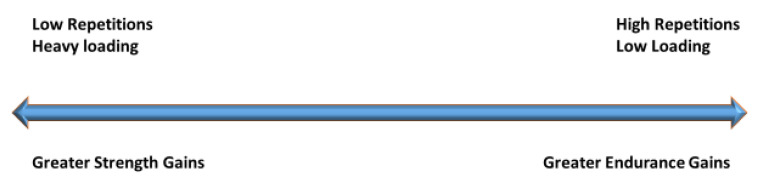
Strength–Endurance Continuum.

1. Strength–Endurance continuum (Figure 1). From a weight-training standpoint, traditionally, this concept indicates that a few heavier loaded repetitions are advantageous for strength development and, higher repetitions of lighter loads are advantageous for developing HIEE [52,53,54,55,56,57].

## 4. Effects of Maximum Strength

The S-E continuum can be assessed in absolute or relative terms. Essentially, as maximum strength increases, then more repetitions and total work can be completed at an absolute load [54]. This occurs largely because a given (absolute) load represents a smaller percentage of the new maximum strength level [52,54] However, when examined on a relative basis in which high intensity endurance is usually measured by repetitions accomplished at the same relative intensity (% 1RM), little change occurs in the number of repetitions performed at the increased absolute load [54]. Thus, it becomes discernible that maximum strength as measured by 1RM plays an important role in altering HIEE. This is apparent, for both absolute and relative tests, in that gains in maximum strength can allow more absolute work to be accomplished. However, it should also be noted that training with low repetitions and heavier weights generally produces superior gains in maximum strength (1RM), so athletes training in this manner may be at a disadvantage when HIEE is assessed using a relative (% 1RM) method as a result of using a substantially heavier absolute load [54,58].

The degree to which resistance training induced gains in isometric maximum strength is related to alterations in the 1RM or HIEE is not completely clear, particularly among initially untrained or minimally trained subjects. It does appear that maximum isometric strength among strength-power athletes chronically training with complex movements (weightlifters and throwers), and well-trained subjects, are altered in accordance to loading demands and tends to increase as the athlete improves their sport performance [15,46,59,60,61].

## 5. Effects of Volume

Although there is general agreement that optimal maximum strength gains require heavy loading, there is little agreement on the S-E continuum at the endurance end. Indeed, the observation of the relatively strong association of maximum strength with HIEE seems to obviate the S-E continuum, at least in part [58]. However, the degree of effect on HIEE and work capacity may depend upon the difference in the repetition range of the training stimulus: for example, 1–3 repetitions per set with heavy loading versus ≥10 or sets of 5 repetitions versus >20 repetitions per set with relatively light loading [54,62].

One reason for the gains in HIEE may be the effect of the total volume of work during training and not simply the number of repetitions per set [9,58].Therefore, another factor that impacts the development of HIEE is the manner in which the training volume is achieved. Obviously, using reasonable loading, the volume of work accomplished during 3 × 10 repetitions would be more than 3 × 2 repetitions, and the increase in maximum strength would be expected to be greater with the heavier loading lower repetition range group but the opposite for HIEE and enhanced work capacity [32,54,62]. However, the effects of 5 × 5 repetitions versus 3 × 10 on HIEE may be similar as the total volume of work would be nearly equal or, perhaps depending upon loading, somewhat larger in the 5 × 5 protocol. Thus, achieving more work by adding sets, and perhaps increasing training frequency, likely enhances HIEE.

The effects of volume on HIEE outcomes has been illustrated by McGee et al. [9] In this 7 wk training study, 1 × 8–12 repetitions to failure (N) was compared to 3 × 10, not to failure (H). An intermediate volume group (P) with decreasing repetitions (not to failure) over the 7 weeks was also examined. The relative estimated volume load of the groups was H > P > N. HIEE was measured by improvement for squats with increasing load to failure, and incremental cycle ergometry to exhaustion. The results indicated that gains in HIEE for both squats and ergometry followed the differences in volume. This study suggests that a S-E continuum, based on volume, exists and is somewhat similar to the suggestions of Painter et al. [58,62].

However, to simply add additional sets versus using more repetitions per set to promote greater volumes may produce somewhat different adaptations that would affect alterations in HIEE. It is well known that variation of resistance exercise variables can lead to different acute neuromuscular and metabolic responses [32,63,64,65,66]. Anyone using complex exercises in their training will note through simple observation and experience that sets of ≥10 will produce greater acute metabolic effects compared to lower repetition sets (≤6). In our laboratory (unpublished data), it has been quite evident that sets of ≥10 produce greater oxygen consumption rates and higher lactates compared to sets of 3 and 5 repetitions. Indeed, higher repetitions per set have repeatedly been shown to produce greater acute metabolic perturbations compared to lower repetition sets [67,68,69]. This has occurred even when work has been equalized [70].

Furthermore, the rest period between sets may play a role. This has been illustrated by McCaulley et al. [70]. Comparisons were made between a high volume squats (H) protocol (4 × 10 at 75% 1RM–90 s rest), a strength protocol (11 × 3 at 90% 1RM 5 min rest) and a power group using 8 × 6 jump squats (3 min rest) at body mass. Work was equalized for total volume. The pre-post response to each protocol indicated a unique pattern for testosterone (T), Cortisol (C), and Sex Hormone Binding Globulin (SHBG). Percent change in T, C, and SHBG from PRE to post exercise was statistically greater in comparison to baseline only for the H protocol. The percent of baseline muscle activity (EMG) of the vastus medialis post exercise was statistically greater following the H compared to the S protocol. Interestingly, the authors concluded that statistically significant acute increases in hormone concentrations were limited to H (hypertrophy) type protocols, independent of the volume of work completed. The H protocol also elicited a unique pattern of muscle activity. Resistance training protocols of different intensity, rest periods and repetitions elicit substantially different acute neuroendocrine responses indicating unique physiological stimuli. Again, these observations are supported by the results of several studies indicating greater physiological and metabolic effects of higher repetitions per set and higher volumes of work [67,68,69,71,72,73]. These observations include, lactate, heart rate, various hormones and oxygen consumption. There is also evidence that of the variables manipulated, the volume of work is more important than the rest period for eliciting a greater metabolic effect [63].

Although differences in repetition number (but equal volume) may not directly affect training induced alterations in CSA [58], it appears that they likely will effect differences in HIEE and work capacity, particularly if there is a relatively large difference in repetition number per set generally agreeing with the findings of [53]. Thus, it appears there is reasonable evidence that training with higher repetitions (≥10) per set, for enhancement of HIEE and work capacity results in greater effects compared to lower repetitions per set, particularly if there is a large difference in the number of repetitions [54,62]. It should also be noted that repetitions per set in the range of 8–12 would likely produce a greater strength stimulus compared to repetitions above that range, thus, providing an additional stimulus for enhancing HIEE [54,62].

Additionally, if higher repetitions per set provide a somewhat better increase HIEE and work capacity, it follows that recovery would be superior as well. Interestingly no studies could be found comparing the effects of higher versus lower repetitions on recoverability. However, higher repetitions per set (≥8–12) have been shown to enhance microvasculature, mitochondrial biogenesis and respiratory capability [25]. Lower repetition high loading has not been shown to cause these types of adaptations with equivocal results at best [74,75]. Interestingly there was no statistical difference between three sets of 8–12 RM and 3 sets of 20–25 repetitions [25], indicating that intensity may play a role in these adaptations. Considering the potential cellular adaptations coupled with alterations in marked improvement in aerobic power [53], these results would suggest that higher repetitions per set (≥8–12) may augment recoverability.

## 6. Training to Failure

Another factor to consider is training to failure. There is no evidence that training to failure produces superior (or even equal) gains in strength or power [22,32,58]. However, based on a review of the literature, it is not clear as to the effects of training to failure on HIEE [62].

It is not unreasonable to assume that regularly pushing to achieve more repetitions, through training to failure, may improve the ability to perform more repetitions and, therefore, total work. Indeed. Izqueirdo et al. [76] found that training to failure results in somewhat greater gains in “muscular endurance” among athletes after 11 weeks of resistance training. However, among well-trained rowers concurrent endurance (rowing) and resistance training for 8 weeks not to failure produced superior results in maximum strength, muscle power, the ability to sustain rowing power, and rowing performance compared to training to failure [77].

From a mechanistic standpoint, training to failure has been shown to create greater metabolic perturbations that could relate to greater training induced adaptations in HIEE [66,78,79]. Furthermore, reviews [32,62] and several longitudinal studies suggest that it is possible that slow-twitch Type I muscle fibers are better targeted and relatively developed to a greater extent with higher repetitions and training to failure [22,75,80,81,82], this may be particularly true when slower movement speeds are used [83,84]. Stronger Type I fibers and a larger Type I CSA enhancement could lead to greater endurance. However, on the other hand, training to failure may prolong recovery to enough of an extent to mute adaptations to training, including those associated with improving HIEE [76,78,85,86,87]. More study in this area is obviously necessary.

## 7. Equalizing Work

Another important factor to consider is the common use of equating workload during resistance training to study a phenomena. While equating the amount of work performed may allow a very controlled examination of the effect of intensity or total work it is not ecologically sound. In real life, day to day training, equating work is not performed and would be counterproductive from a time aspect, as it requires considerable time to plan and complete work equated programs. Time is an especially important consideration for an individual sport and individual athlete basis. Furthermore, a potential problem with equating work that has not been explored well, concerns the use of atypical set/repetition protocols. If one assumes that a particular loading scheme is optimum for a specific adaptation then odd combinations may actually interfere with potential alterations in physiology and performance. For example, to examine the enhancement of HIEE of a higher repetitions protocol compared to a lower repetition protocol: based on a squat training session volume load using 5 × 10 at a 60% 1RM (1RM = 100 kg) load would require 11 × 3 at a load of 90% 1RM or 13 × 3 at 80% of 1RM in order to be approximately equal. Although 5 × 10 protocols are commonly performed during accumulation phases among athletes [32,59,60], 11 × 3 repetitions are not typically performed at any time. In our opinion this occurs for a number of reasons relating to time constraints and recovery needs. Although not definitive, evidence in support of optimum loading schemes has been shown recently among trained subjects [88]. In this study, repetitions to failure (RTF) at 70% of 1RM were completed weekly as 12, 18 or 24 sets; sets were equally divided between the back squat and leg-press exercises. The results suggested that the middle dose (18 sets per week) range optimized the gains in back squat 1RM. The results suggest that there may be an optimum number of sets for a given repetition scheme. However, if researchers continue to compare training programming strategies by equating workload they may mask the effects of truly optimal dosages and ratios of volume and intensity for targeting specific physical adaptations.

## 8. Summary and Conclusion Strength-Endurance Section

**Figure 2:**
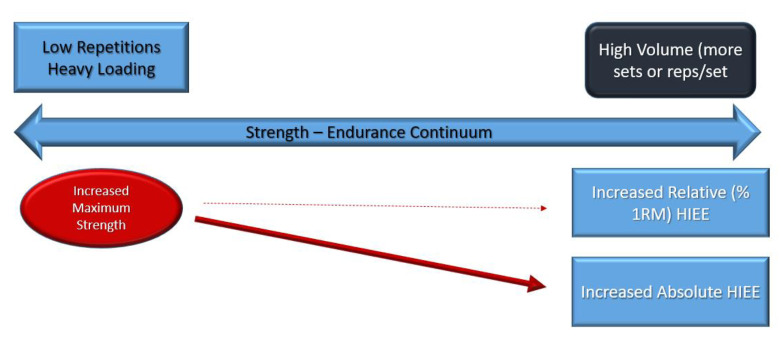
Training: Equalized Work Volume.

**Figure 3:**
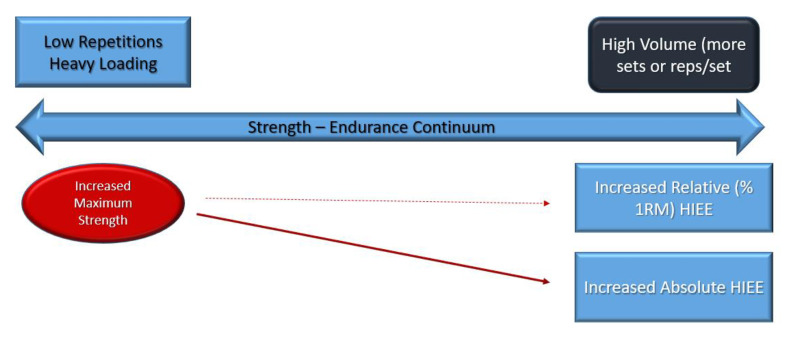
Training: Non-Equalized Work Volume.

Thus, we believe based on current available evidence a strength–endurance continuum does exist on an absolute basis. This is considering the following factors (Figure 2 and Figure 3):

- Higher repetitions per set produce higher metabolic stress driving potential metabolic alterations resulting in greater HIEE and expanded work capacity
- The potential for better recovery as a result of the greater metabolic alterations with higher repetitions per set
- The continuum is ecologically sound
- The continuum provides part of the basis for periodization protocols [32]

2. Dynamic Correspondence (DC)

The aims of resistance training deal with the positive exploitation of immediate, accumulative, long-term and delayed effects of imposed training demands [32,50,89,90,91,92] for the enhancement of sport performance. These types of performance alterations, particularly long-term, depend on the organization, sequencing, and manipulation of the basic training principles–overload, specificity, and variation [32,50]. Although mechanistically complex, transfer of training effects (ToTE) is of paramount importance for athletes and coaches, as strength should be developed within the context of the sport to maximize its effectiveness [32,50].

**Figure 4:**
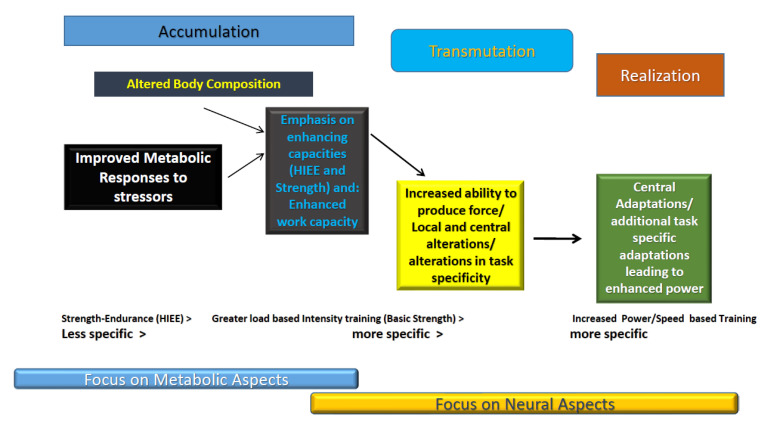
Sequential Phase Based Approach to Metabolic and Neural Alterations.

Most coaches use some type of periodization methodology [32] Typically, as training moves from extensive (accumulation) to intensive (transmutation and realization) considerations in workload, it also shifts from general to more specific (Figure 4). During transmutation and realization (special preparation and competition phases) [32] periods, ToTE is particularly important and appropriate assessment of the ToTE to sport performance becomes particularly valuable. Even within the principle of specificity, it may be necessary for the coach to consider more nuanced factors of sport specificity beyond the obvious and somewhat superficial metabolic and mechanical aspects. A more nuanced, deeper consideration of ToTE, *Dynamic Correspondence*, was created by Yuri Verkhoshansky in the early 1990′s [50,93,94]. Dynamic correspondence is concerned with logically connecting various aspects of training specificity into more quantifiable, directed components. These aspects include the:

(1) amplitude and direction of movements,
(2) accentuated regions of force production,
(3) dynamics of effort,
(4) rate and timing of maximum force production, and
(5) arrangement of muscular work.

## 9. Amplitude and Direction of Movement

Two of the most evident aspects of specificity (dynamic correspondence) are the amplitude and direction of movement. Amplitude of movement refers to the range of motion (ROM) or degree of movement displacement. For instance, rowing and bench pressing movements have somewhat similar amplitudes but occur in opposite directions [50,93]. One can argue that the direction of an exercise is the most widely accepted form of specificity. However, the directions that forces are actually applied in during specific movements are not always clear. For the context of this discussion, it is important to note that in typical sport situations, independent of the direction the athlete moves, forces in sport are often initiated by applying force through the ground. As a result, any training exercises that are initiated differently such as open kinetic chain exercises are not likely to transfer to the same degree even if the muscle groups used are similar [95,96,97,98,99]. Furthermore, even relatively small changes in the direction of a movement in relation to the body, such as bench pressing with different grip widths or at different angles alters the activity of the muscle groups used [100,101] and may affect transfer to movements like throwing (i.e., shot put, discus) at various angles. Enhancement has been noted for measures of maximum dynamic and isometric strength, explosive strength (RFD) as well as running and jumping have been noted with the use of training squats of varying amplitude [102,103,104]. Evidence also indicates that using exercises with larger amplitudes may augment positive effects on sport movements by better mimicking sport-specific amplitudes [105,106].

Considering the direction of force application, a distinction must be made between the global frame of reference and the athlete frame of reference [107]. For example, during the acceleration phase of a sprint, an athlete produces large amount of horizontal force [108,109] relative to the global frame. A more horizontal force vector is achieved by the alteration in the athlete’s posture by leaning forward fore. Therefore, relative to the athlete, force is applied through the longitudinal axis of the body, thus in a vertical direction [107].

Indeed, -semi-ballistic exercises such as weightlifting lifts (snatch and clean) and derivatives and partial squats share many similarities between the knee, hip, and torso angles as well as the total amplitude that occur in athletic movements such as sprinting [104,110]. Squatting and weightlifting movements can increase vertical force producing capabilities similar to those required for jumping, leading to an increase in jump performance [111,112] and sprint performance [107,113], while open chain exercises that do not develop these vertical abilities have shown minimal transfer [95]. Both full and partial ROMs movements have shown a transfer of training effect. However, full ROM training appears to develop qualities underlying sport performance such as muscle cross-sectional area (CSA) and potentially strength to a greater degree than only partial movements [110,114]. These muscular adaptations in “capacity” can provide athletes a greater capability to develop force thus possibly increasing their potential to benefit from more specific training in later phases. Considering the available evidence, exercises that develop ground reaction forces using both partial and full ROMs in training should be advantageous [50,103]. Therefore, logically, training should progress from less specific to more specific amplitudes reflecting the sporting actions. To accomplish appropriate progressions it is necessary that coaches gain an understanding of the joint angles and amplitudes most commonly used in their specific sport, and appropriately choose exercises that develop them [50].

## 10. Accentuated Regions of Force Production

Accentuated regions of force production deals with the specificity of muscular effort and consequently force application alterations throughout the course of a movement [93] Although few studies have directly studied the diverse regions of force production for different exercises [103], this concept may be a possible explanation of why certain exercises have shown a greater transfer to athletic movements than others [50].

Explosive ballistic type training with high RFD’s appears to be one of the most effective modes of resistance training to improve athletic performance [19,115,116]. Ballistic type training has produced increases in vertical jump height in elite volleyball players [117,118], as well as improvements in both throwing and base running speed in baseball players [119,120].

Part of the reason that ballistic training can transfer well to athletic performance may partly be due to similarities in the accentuated regions of force production. For example: accentuated regions of force production have been shown to occur during the stance phase of sprinting [121,122], as well as the braking and propulsive phases of jumping [123,124]. Ballistic movements require acceleration of a mass through the entire range of motion [115,125], as a result ballistic movements appear to share more similar accentuated regions of force than traditional resistance training that require end movement deceleration. Indeed, Newton and Kraemer [126] have presented evidence indicating that the force curves of the ballistic exercises such as a bench throw were more similar to typical athletic movements. However, it is important to note that improvements in athletic movements have also been observed resulting from substantial gains in strength [127] and traditional resistance training [128]. However, it appears that a combination of heavy non-ballistic and ballistic training can result in greater increases than either in method in isolation, especially when dealing with well-trained athletes [16,111,126,129]. It should also be noted that evidence indicates that weightlifting movements, which produce both high forces and are semi-ballistic in nature can produce substantial gains in ballistic movements such as sprints and vertical jumps including recreationally trained subjects [130] well-trained athletes [131]. Indeed, weightlifting movements, compared to ballistic training or powerlifting training have been shown to produce superior improvements in sprinting, change of direction and vertical jump among physically active college students [132], high school American football players [112] and female volleyball players [133].

Recently, different means of manipulating the accentuated regions of force production have been explored with varied success. Using free weights, direct manipulation force, including the accentuated region of an exercise can result through the use of adding devices such as elastic band (EBR) tension or weight releasers (WR). EBR is a training method that has been used previously in an attempt to maximize force production by accommodating for natural strength curves [50] EBR peak resistance occurs at the very end of the range of motion where the band is stretched the furthest, which results in regions of force production dissimilar from both the traditional exercise [38,134,135] and typical athletic movements [38,136]. Indeed, examination of the research dealing with transfer of training resulting for EBR training, there has been little evidence that it increases jump performance as well or beyond that of traditional training methods [137] even if it may increase measures of strength and power [134,138,139]. One potential consequence of this outcome is that the regions of force production being developed when training with EBR are substantially dissimilar those used during typical athletic movements resulting in alteration of the natural coordination of the movement [38,136]. Alternatively, accentuated eccentric loading (AEL) may allow manipulation of the accentuated regions of force production such that the natural movement patterns are augmented positively. By overloading the eccentric phase of a movement and then suddenly removing the load at the in intimation of the concentric portion, in a manner possible with the use of weight releasers, early concentric force may be potentiated. By using AEL, force production, including the accentuated region, of force production may be increased to a greater degree than in normal loading [140,141,142]. The degree of transfer to athletic performance remains to be seen.

## 11. Dynamics of Effort

The ability to apply or withstand varying magnitudes of force at different movement velocities is paramount for superior athletic performance [50]. Dynamics of effort concerns the force-velocity characteristics of training means and how they relate to specific athletic movements. Thus, during specific sport movements, the dynamics of effort of training should encompass the associated force magnitudes as well as movement and contraction velocities [50].

Supporting this concept, evidence demonstrates that heavy-load resistance training produces larger increases in maximal strength compared to low-load [32,143]. However, low-load higher velocity training may be necessary for improving high-velocity athletic performance, particularly among well-trained athletes [32,144,145,146,147]. Combined strength and power training seems to be the most effective strategy for improving performance in athletic movements [15,16,148]. However, it should be noted that high-load resistance training can be more effective at improving many athletic performances in weaker athletes than power training [149,150], indicating that a foundation of strength may be necessary to optimize power development. Specific periodized training models (e.g., block periodization) have been shown to accomplish this the development of appropriate foundation through the use of phase potentiation in which specific athletic qualities such as strength-endurance, strength, power, and speed are varied in emphasis and are sequenced in a manner aimed at enhancing subsequent phases [32,146].

Additionally, evidence indicates that purposeful rapid muscle contraction and movement plays an important role in improving movement velocity [151,152]. Thus, if an increase in movement velocity is the desired goal, athletes should execute rapid movements with conscious intent. Coaching attention should be focused on athletes, encouraging them to perform all sets, especially work and down sets, with maximum intent to move with explosiveness and high velocity throughout the entire movement. This can result in maximizing the stimulus of both heavy and light loads and the various forces and velocities produced within a single training session without any change to the overall training volume [50].

## 12. Rate and Time of Maximum Force Production

For most sports, a positive outcome can be determined by the ability to maximize force production during critical time intervals. This ability results from being able to generate greater force within a certain time frame (i.e., increased rate of force development). Therefore, training stimuli should be organized to enhance the rate of force development (RFD) and use tasks that may have a similar time constraints to sports specific movements. For example, different jump tasks can be characterized based on ground contact times as utilizing either a slow or fast stretch-shortening cycle (SSC) [153], which differ mechanistically [154,155]. Therefore, coaches should be selective in their selection of training protocols in order to ensure transfer to athletic movements. Movement characteristics such as ground contact time and SSC duration can also reflect kinematic differences that could affect performance outcomes [121,153]. This observation indicates that coaches should also focus on proper technical execution during training to promote correspondence of rate and time of force application [50]. Furthermore, the shape of the force-time curve may be related to sprint and jump performance [121,124]. This observation provides additional evidence that reasonable skill level and precision of movement are necessary to ensure the development of an appropriate kinetic profile corresponding to sports specific movements. Therefore, coaches should consider factors such as ground contact time, movement duration, and stabilizing superior exercise technique when addressing the correspondence of the rate and time of force application [50].

Considerable thought should be given the multiple adaptations that may contribute to improvements in rate of force development. Reviews of the literature [156,157] call attention to both neural and muscular determinants of explosive strength (RFD) indicating that both heavy resistance, as well as high power and plyometric training can produce favorable changes in motor unit recruitment and discharge rates that contribute to RFD. Importantly, considerable evidence indicates that rapid ballistic contractions can result in positive adaptations in motor neuron discharge rates that contribute to increases in RFD, particularly during the early rise of RFD [156,157,158,159]. In part, improvement of RFD through high-load resistance training is a result of the neural targeting and the resulting hypertrophy of type II muscle fibers and morphological changes of whole muscle [160,161]. Augmenting tissue stiffness. Including the tendon may also increase force transmission leading to a greater RFD [162]. Different modes of training may act differently to affect adaptations in the tendons [162]. For example, long-term training adaptations in the lower limb (e.g., patellar and Achilles tendons) appear to affect running performance and are markedly different between sprinters and endurance athletes [163]. Therefore, from this aspect, in order to take full advantage of dynamic correspondence, it is quite important to be aware of the “scalability” of the long-term training [50] –long-term programming aimed at specific adaptations and underlying mechanistic alterations that can support a more substantial total training effect and performance outcome.

## 13. Regime of Muscular Work

The regime of muscular work distinguishes the type of muscular contraction. For example, regimes may be classified as concentric, isometric, eccentric or those with stretch shortening cycles (SSC). Note that SSC’s may or may not include rhythmic, cyclical action, typical of walking and running. Muscle actions (i.e., concentric, isometric, eccentric) rarely happen in isolation in sport. Athletic movements are typically characterized by some form of SSC. However, utility of this component of dynamic correspondence has been questioned due to the interdependent and complex nature of force production in sport [164]. When considered separately, concentric actions appear to be more sensitive to the specificity of kinetic and kinematic properties of contraction [165]. However, eccentric training appears to have a broader effect on a continuum of force outputs and velocities [165]. This indicates that different adaptations take place between concentric and eccentric actions [50,90].

Although, the concept of specificity as it pertains to different types of muscle contraction is well-established [166,167], there are unique aspects which can effect subsequent adaptation between concentric and eccentric actions [168]. For example: superior mechanical efficiency and energy dissipation have been observed during eccentric contractions, particularly submaximal, compared to concentric contractions [169,170]. Furthermore, different structural alterations have been observed as a result of training studies comparing concentric and eccentric contractions. Greater muscle hypertrophy and inhomogeneous hypertrophy, which may be linked to a variety of performance outcomes, appears to differ depending on contraction type. For example: concentric training appears to have greater influence on the muscle belly compared to eccentric training, which appears to influence the distal portion of the muscle to a greater degree [171,172]. Interestingly, when workload is equal, similar structural adaptations can occur, even though hypertrophic changes appear to be achieved by distinct structural alterations, which may be regulated by different myogenic and molecular responses observed between eccentric and concentric contractions [172] Additionally, most studies have shown eccentric training to preferentially increase type II muscle fibers [172]. Furthermore, eccentric training has been shown to increase fascicle length, while concentric training has been more associated with increases in pennation angle [171,172,173,174,175]. Importantly, alterations in tissue morphology can influence the development of subsequent physical capabilities and are important considerations when designing programs.

A unique coaching situation is presented when considering SSC and complex athletic actions. Consideration must be taken of the specific mechanisms associated with each type of contraction independently, as well as together. Most reviews and studies of muscle contraction, including during resistance training support an orderly size dependent motor unit recruitment [176,177,178,179,180]. However, the potential for eccentric actions to violate the size principle has been demonstrated [181], meaning that while clear and predictable activation patterns may exist concentrically, a different pattern of recruitment may emerge eccentrically, particularly at faster velocities of contraction. When considering eccentric and concentric actions as a pair, as is typical in the training and actions of athletes, a complicated sequence of neural control strategy appears to be occurring within a given movement [90]. This is further illustrated as a result of different discharge rates and activation thresholds of motor units involved in eccentric contraction compared to concentric [182,183,184].

While it may seem logical, to regularly program complex SSC movements that appear similar to sporting action, the coach must also consider athlete development (capacities). Development concerns several facets dealing with structure, metabolism and neural aspects. Importantly, the machinery (i.e., involved muscles, tendons, etc.) must be robust enough to handle the high-stress nature of these complex often high intensity actions. Therefore, a developmental process in which capacities are enhanced may allow exploitation this aspect of dynamic correspondence across a spectrum of emphasis. Early in general preparation (accumulation), evidence indicates that choosing to exploit contraction-specific mechanical loading to make targeted changes to the muscles and tendons and to build the overall capacity of the athlete is reasonable and can potentiate further long-term outcomes [32,147,185]. As training progresses, the coach may then aim to turn attention towards the uniqueness of neural strategies and adaptations to maximize sport potential and minimize the risk of an injury to the athlete [32].

## 14. Additional Considerations

In addition to the dynamic correspondence criteria discussed so far in this review, Goodwin and Cleather [164] suggested adding a sixth, which deals with segmental interrelation. This 6th criterion suggests coaches should account for the complex interrelationship between global (body), segmental (joint), and muscular actions during athletic movements. This idea infers that coaches should consider the criteria of dynamic correspondence both as individual criteria as well as collectively in order to develop training programs that readily transfer to sport. Considerations should also be given to the type of transfer to sport. Training strategies can directly transfer to improving performance related variables such as running, jumping, throwing, or change of direction performance. Furthermore, they may indirectly transfer by developing capacities they would affect a more durable athlete that is less likely to get injured [186,187] and more likely to benefit from training [188].

Coaches must have a strong and clear understanding of the kinetic and kinematic relationships between specific training strategies and athletic performance. Dynamic correspondence principles must also be considered when taking into account the more general training principles of overload, specificity, and variation [32]. For example, certain exercises may fit a majority of the criteria covered, but if they are not properly loaded and sequenced over time and varied throughout the training plan, they may not result in an appropriate transfer of training effect [32]. One common factor often observed and when examining research, is that exercises with greater specificity to sport movements, such as ballistic and plyometric training, are inclined to transfer to a greater degree in stronger athletes [32,146,189]. Therefore, relinquishing heavier loads in an attempt to make an exercise more specific to certain sport movements may decrease the development of appropriate capacities and ultimately potential transfer of training effect, particularly in athletes that are relatively weak or are not well-trained. Although, overall potential of a particular exercise or training strategy to promote transfer to sport is often difficult to assess. Use these criteria of dynamic correspondence can aid coaches in evaluating what training strategies may be the most beneficial, and how they should be sequenced into the training plan [50].

## 15. Summary

Perhaps the most important of the training principles deals with *Specificity*. Our review represents arguments that specificity has two major components. A strength-endurance continuum (S-EC) and adherence to principles of Dynamic Correspondence. We believe the available evidence does substantiate existence of this continuum from two aspects. Indeed, the S-EC exists, particularly if work is equated as a high load low repetition scheme at one end (strength stimulus) and high volume (HIEE stimulus) at the other. However, there is also some evidence that supports the continuum as a repetition paradigm with high-load, low repetition at one end (strength stimulus) and a high repetition, low load at the other end. The second paradigm is most apparent under three conditions: (1) ecological validity—in the real world, work is not equated, (2) use of absolute loads in testing and (3) a substantial difference in the repetitions used in training (for example 2–5 repetitions versus ≥10 repetitions).

Additionally, adherence to the principles and criteria of dynamic correspondence can allow for greater transfer from training to performance measures. Typically and logically, in order to optimize transfer, training athletes requires a reasonable development of capacities (i.e., structure, metabolism, neural aspects, etc.) before more specified training takes place.

## Data Availability

Not applicable.
